# Book Reviews

**Published:** 1888-11

**Authors:** 


### Book Reviews.

**Reports from the Patho-Biological Laboratory, University of Nebraska.** By DR. FRANK S. BILLINGS, Director. First Report, Southern Cattle Plague and Yellow Fever; pp. 141. Second Report, Swine Plague; pp. 414. Lincoln, Neb., 1888.

The rapidity of movement in these times of ours is truly surprising. A boy is born, grows up, and before he finishes college a new State has sprung up, is admitted into the Union, and its senators and representatives are discussing upon the floors of Congress the great problems of finance and political economy; its schools and colleges are established, and even universities are created, which are sending out its scientific publications before this same boy reaches the mid-noon of manhood. The University of Nebraska is an example of this sort, illustrating with a verity the rapid and substantial progress of American thought and action. These two reports demonstrate not only the capabilities of their author, but the important part the State should play in the great problems of preventive medicine. Work of this nature can only be accomplished by a liberal outlay of money, such as no private institution could afford, unless endowments are dealt out with the generous hand of a Johns Hopkins. These, however, happen so seldom, that it is only through national or state appropriations that the work can be accomplished with that promptness which keeps pace with its necessity. The first report is one of great interest to the sanitarian, public health officer, and the quarantine commissioner. If it can be shown that yellow fever, or any other disease, may be propagated by the transportation of diseased cattle from one part of the country to another, then the quarantine authorities must enforce the most rigid inspection of cattle in transit, and insist upon their thorough sanitary care during travel, as well as the prolonged quarantine of suspicious animals. The second report will be found of great value to the swine-growing interests of the country, and must awaken a general interest in the cause of swine sanitation amongst the pork-packing capitalists of the country, whose vast interests are at stake The illustrations at the close of the text in this report are especially to be commended for their clearness of execution and thoroughness of detail. While there is so much to be said in praise of these reports, we cannot close this notice without recording one or two points which mar their scientific *ensemble*. The illustration of the director's laboratory as a frontispiece in the second is a matter of questionable taste, particularly as there are no especially novel or distinctive features brought out thereby. Then, too, we would wish that less space had been given to mere controversial matters. To be critical is well; to be hypercritical is not always desirable in a scientific publication. The press-work and paper of the volumes are all that need be desired; we wish, however, they were more substantially bound.

---

**Transactions of the Medical Association of the State of Missouri, at its Thirty-first Annual Session. 1888.**

**Transactions of the Medical and Chirurgical Faculty of the State of Maryland. Ninetieth Annual Session. 1888.**

In the examination of these two volumes, one the transactions of a representative State medical society of the East, and the other of the West, one becomes impressed with the valuable practical work done by State medical societies at their annual sessions. The transactions of the Missouri society contains forty-one papers on professional subjects, covering the field of the various practical departments of medicine. Does one wish to examine tables of gunshot wounds of the abdomen in which laparatomy has been performed, he will find them here carefully prepared by Dr. H. C. Dalton, of St. Louis, who reports in detail a successful case of his own. Dr. N. B. Carson, of St. Louis, discusses the question of Abdominal vs. Lumbar Colotomy in an interesting paper, based upon three cases of his own, and in which he groups the results obtained by other operators after the several methods of Amusat, Littre, and Callisen. Other papers, involving obstetrical, ophthalmological, genito-urinary, sanitary, and neurological medicine, besides still others discussing questions in general medicine and surgery, go to make up a most interesting volume of 462 pages.

Turning to the transactions of the Maryland society, we find much interesting material embraced in the reports of the various sections into which this society divides itself, besides the voluntary communications offered afterwards. Among the latter, Dr. J. H. Branham, of Baltimore, reports a case of complete excision of the larynx for epithelioma. Though this case did well for three days, it succumbed to pneumonia on the fourth, having been weakened by previous hemorrhages to such an extent as to greatly complicate the general and local conditions. It is to be regretted that these books were not offered in some sort of stiff binding, no matter how simple, for no one in this busy age likes to carry paper-covered volumes in his library. A well-prepared index, too, would add greatly to their convenience and value.

---

**Disinfection and Disinfectants:** Their Application and Use in the Prevention and Treatment of Disease, and in Public and Private Sanitation. By the Committee on Disinfectants appointed by the American Public Health Association. Concord, N. H. 1888.

At the present day it is essential that every physician, no matter whether practicing general surgery, general medicine, or a specialty, shall make himself familiar with the principles of disinfection, and with the action of disinfectants. This book furnishes just the information that every well-informed physician must needs have, and without which he cannot either successfully combat disease or preserve the health of his clients. It is prepared by a committee of experts, every one of whom may be considered authority upon the subject of which he treats. Much of it is the result of original experimental investigation, which has occupied the committee upwards of three years in perfecting, or reducing to a sufficiently practical basis for authoritative publication. It is in this book that one may ascertain the relative value of the various commercial disinfectants that are offered to an unwary public—information that may prevent the disastrous consequences that result from reliance upon an inadequate germicide or disinfectant.

The chairman of the committee, Dr. George M. Sternberg, says in his report on "Commercial Disinfectants," page 12, that "one agent advertised as a 'germicide' *par excellence*, * * * failed, *after two hours'* exposure, to kill the organisms in our test solution in the proportion of twenty per cent. Yet this fluid is, by some contrivance, to be thrown into the water-closet of every germ-fearing citizen when he pulls the handle, so that it may catch the germs on the fly, and extinguish their power for mischief before they reach the sewers."

The work further treats of chemical disinfectants and germicides, methods of practical disinfection, disinfection by moist and dry heat, disinfection of clothing and apartments, ships, railway cars, etc. It would be interesting to pursue this subject in detail, but our space forbids, and we must content ourselves at present with this brief reference to a most valuable contribution to the literature of an all-important subject, for the first time grouped in such a practical form. A compilation of the bibliography of the subject from 1880 to 1888, by Dr. George H. Rohé, the secretary of the committee, and a complete index prepared, we presume, by Dr. Irving A. Watson, the Secretary of the American Public Health Association, together with fine paper, good press-work, and a neat substantial binding, serve to make the volume attractive, durable, and useful.

---

**Diet Tables.** REED & CARNRICK, 447 and 449 Greenwich street, New York.

These most conveniently arranged leaflets, grouped and bound together in morocco, supply the physician with the ready means of answering many questions in regard to diet in disease, and in a form that will be more likely to receive attention than mere verbal directions. They have been carefully prepared by eminent and experienced physicians, and may be accepted as reliable and authoritative guides in the dietary management of diseases and diatheses. The publishers furnish them gratuitously upon application.

